# Soft partial release of non-aggressive stent retriever technique for very distal arterial occlusion stroke

**DOI:** 10.1177/15910199241299471

**Published:** 2024-11-25

**Authors:** Peter B Sporns, Mohammad Almohammad, Zoltan Puskas, Hassan Soda, Thi Dan Linh Nguyen-Kim, Ole Simon, Lars Timmermann, André Kemmling

**Affiliations:** 1 Department of Neuroradiology, 30262 University Hospital Basel, Basel, Switzerland; 2 Department of Diagnostic and Interventional Neuroradiology, 37734 University Medical Center Hamburg-Eppendorf, Hamburg, Germany; 3 Department of Radiology and Neuroradiology, 31012 Stadtspital Zürich Triemli, Zürich, Switzerland; 4 Department of Neuroradiology, 61061 University Hospital of Giessen and Marburg, Campus Marburg, Marburg, Germany; 5 Department of Neuroradiology, 39515 Rhön Klinikum, Campus Bad Neustadt, Bad Neustadt an der Saale, Germany; 6 Department of Neurology, 39515 Rhön Klinikum, Campus Bad Neustadt, Bad Neustadt an der Saale, Germany; 7 Department of Neurology, 61061 University Hospital of Giessen and Marburg, Campus Marburg, Marburg, Germany

**Keywords:** Stroke, thrombectomy, distal, DVO, EVT

## Abstract

**Background:**

Endovascular thrombectomy (EVT) for very distal vessel occlusion (DVO) stroke is increasingly performed but there is insufficient evidence on the efficacy and safety of distal EVT techniques. We hypothesized that the technique of soft partial release of non-aggressive stent retrievers (SPORNS) reduces friction on the perforating vessels during thrombectomy and thereby reduces bleeding complications.

**Methods:**

Retrospective study including consecutive DVO patients who were treated with the SPORNS technique between 1 January 2022 and 31 December 2022 at two tertiary stroke centers. DVOs were defined as isolated occlusions of the M3 and M4 segments of the middle cerebral artery, occlusions of the A2 and A3 segments of the anterior cerebral artery, and occlusions of the P2 and P3 segments of the posterior cerebral artery or of the superior cerebellar artery. The technique is described in detail and procedural and clinical outcomes are given.

**Results:**

Twenty-four patients were treated with the SPORNS technique of whom 22 (92%) had complete or near complete recanalization (eTICI 2c/3). National Institutes of Health Stroke Scale (NIHSS) decreased from a median of nine (IQR 7–13) at admission to three (1–5) at discharge and 18 patients (75%) achieved a good outcome (modified Rankin scale 0-2) at day 90 post-stroke. Two patients (8%) had a small subarachnoid hemorrhage and two patients (8%) had a symptomatic intracerebral hemorrhage on follow-up imaging.

**Conclusion:**

For the treatment of very distal arterial occlusions, the SPORNS technique employing a soft partial release of a non-aggressive stent retriever is safe and effective for the thrombectomy of small clots. The technique potentially yields a lower rate of subarachnoid hemorrhages while achieving an excellent rate of complete and first-pass recanalization.

## Introduction

Over the past years, several randomized trials have proven the safety and efficacy of endovascular treatment (EVT) in large vessel occlusion (LVO) stroke.^1^ Whereas around 35%–40% of acute ischemic strokes occur due to LVO, 25%–40% are caused by medium-vessel occlusions and very distal vessel occlusion (DVO).^2–4^ Due to the more distal occlusion location and less extensive ischemia, it is commonly assumed that clinical outcomes after DVO are favorable compared with LVO strokes. However, cohort studies suggest that outcomes are frequently poor after DVO, despite the best medical management.^5^ Given the high efficacy of EVT in LVO stroke and the substantial morbidity associated with distal vessel strokes, EVT is now increasingly performed for these occlusions. Many neuro interventionalists already routinely offer EVT for isolated M2 occlusions,^6^ but there is currently no high-level evidence for EVT in more distal arteries, and it is likely that due to the smaller vessel size, EVT may yield marginal clinical benefit at the cost of increased risk of procedural complications.^7^ The smaller caliber of more fragile distal arteries and the more distal, thus harder to reach, occlusion location compared with LVOs warrant changes in EVT technique and technology to minimize risks. A recent in-vitro study has shown that arterial collapse frequently occurs during aspiration thrombectomy and is more likely to happen in smaller arteries,^8^ which may have implications for thrombectomy techniques such as the recently described microcatheter aspiration technique.^9^ Moreover, several studies have shown that stent retrievers have different radial forces and friction depending on their design and diameter.^10–12^ Thus, using a stent retriever in very distal occlusions may result in high friction leading to critical pulling forces and shearing of the perforating arterial branches resulting in higher rates of subarachnoid hemorrhage. We hypothesized that our proposed simple method (the soft partial release of a non-aggressive stent retriever) reduces these critical forces minimizing complications by preventing the shearing of perforating arteries while maintaining effective recanalization. Thus, our aim was to evaluate the safety and efficacy of this recanalization technique for DVO strokes.

## Methods

### Study design

This is a retrospective study including consecutive acute ischemic stroke patients caused by a DVO who were treated with EVT. All patients who were treated employing the SPORNS technique were included between 1 January 2022 and 31 December 2022 at two tertiary stroke centers. DVOs were defined as isolated occlusions of the M3 and M4 segments of the middle cerebral artery, occlusions of the A2 and A3 segments of the anterior cerebral artery, and occlusions of the P2 and P3 segments of the posterior cerebral artery.

In general, the individual method of treatment was chosen at liberty by the treating interventionalist without any prior specification or intent. Reasons for performing EVT were that endovascular access was judged possible by CTA, the National Institutes of Health Stroke Scale (NIHSS) was at least two, or if there was a disabling deficit (i.e. hemianopsia for PCA stroke and aphasia), and if there was a mismatch between ischemic changes on non-contrast CT and potentially salvageable tissue on CTP and/or expected clinical deficit.

The study was approved by the ethics committees of the participating centers with the waiver of informed consent for the evaluation of retrospective data. All patients underwent flat detector computed tomography (FDCT) at the end of the intervention and follow-up imaging within 24–48 h after thrombectomy.

### Outcomes

The primary outcome was complete or near complete recanalization (according to the extended thrombolysis in cerebral infarction [eTICI] scale 2c/3)^13^ at the end of the procedure, considering the territory distal to the targeted occlusion as 100%, reperfusion rates were translated into the eTICI grades.

Secondary efficacy outcomes included good clinical outcomes at 3 months, defined as a modified Rankin scale (mRS) of 0-2, and excellent clinical outcomes (defined by a mRS of 0 or 1). Safety outcomes included the rate of complications, postinterventional subarachnoid hemorrhages (SAH), and cerebral hemorrhage (of any type according to the European Cooperative Acute Stroke Study [ECASS]).

### Technical description of the SPORNS technique

The technical rationale aims at minimizing the risk of hemorrhagic events during thrombectomy in DVO. We assume, that a substantial reduction of pulling forces on small perforating arteries by the stent-retriever may limit hemorrhagic complications. To achieve this, a stent retriever design with limited radial force is chosen, while being unsheathed partially at the microcatheter tip to further reduce pulling forces by friction during thrombectomy. The thrombectomy procedure for DVOs generally starts by gaining access to the cerebral vasculature usually with an eight French (F) guide catheter or a long eight  F sheath distally in the internal carotid artery (ICA) or in cases of a posterior circulation stroke with a 6F or 7 F guide catheter in the vertebral artery. A conventional coaxial system is used to gain access to the DVO. It consists of a soft intermediate catheter or aspiration catheter (i.e. Sofia 5f, Microvention, Tustin, CA, USA) and a 0.021-inch microcatheter (i.e. Headway Duo or Trevo Pro 14, Stryker Neurovascular, Fremont, USA) which is advanced past the occlusion site with the help of a 0.014-inch microwire (i.e. Synchro 2 support, Stryker Neurovascular, Fremont, USA).

**Figure 1.**
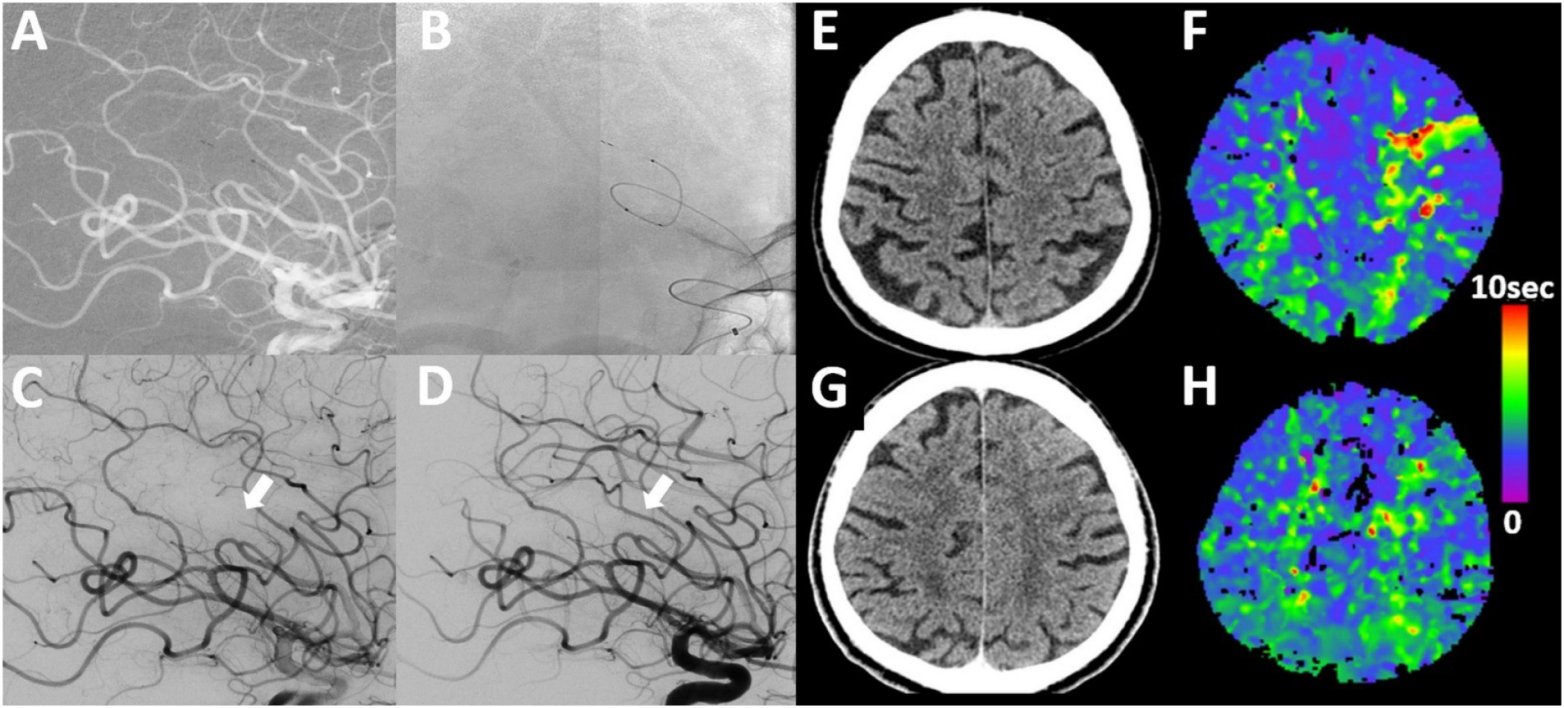
Example of SPORNS technique in a patient with M4 occlusion. A male patient presenting with NIHSS 5 (right-sided weakness and incomplete aphasia). (A) Lateral DSA of distal M4 occlusion which was not seen on CT-angiography but CT-perfusion without early infarct lesion in CT (E, F (TMax-map)). (B, C) Thrombectomy employing the SPORNS technique with partial release of the stent-retriever (pRESET lite 3–20 deployed through SL 10 microcatheter), resulting in complete recanalization (D). On 24 h follow-up, there are no signs of infarct or hemorrhage in non-contrast CT and CT-perfusion shows no signs of previous cortical ischemia in the left hemisphere (G, H).

SPORNS: soft partial release of non-aggressive stent retriever; NIHSS: National Institutes of Health Stroke Scale; DSA: digital subtraction angiography; CT: computed tomography.

A stent retriever with a 3 mm diameter and low radial force (Phenox pRESET lite 3 × 20 mm, Phenox, Bochum, Germany) is then advanced to the microcatheter tip. The microcatheter is then retracted until 20%–30% of the total length of the stent-retriever is unsheathed with the thrombus covered by the distal unsheathed part of the stent retriever (Figure 1). Finally, the stent retriever and microcatheter are slowly pulled back simultaneously into the aspiration catheter that is positioned in the M1 segment of the MCA or basilar artery. Aspiration is only started when the stent retriever has reached the tip of the aspiration catheter to prevent collapsing of the artery and complications.^8^

### Statistical analysis

Standard descriptive statistics were applied for all displayed data. Continuous variables are presented as medians and interquartile ranges (IQR). All statistical analyses were performed with SPSS Version 25 (IBM Corporation, Armonk, NY) and Stata 17.0 (StataMP, StataCorp, TX).

### Data availability

The data that support the findings of the study are available from the corresponding author, upon reasonable request.

## Results

### Patient characteristics

A total of 24 patients were included in our study, of whom 11 (46%) were occlusions of the M3 segment, one was an M4 segment occlusion (4%), five were occlusions of the anterior cerebral artery in the A2 segment (21%), six were occlusions of the posterior cerebral artery (5×P2 segment and 1×P3 segment; 25%) and one was occlusion of the superior cerebral artery (SCA, 4%).

The median age was 73 (IQR: 62–87) and eight patients (33%) were female. The mean time from onset to groin puncture was 183 min (IQR: 112–245). Mean ASPECTS at baseline imaging was nine (IQR: 8–9), median NIHSS on admission was 9 (IQR: 7–13) and 13 patients (54%) received additional intravenous thrombolysis (Table 1).

**Table 1.** Baseline characteristics.

Baseline characteristics	All (*n* = 24)
Age, median (IQR)	73 (62–87)
Female sex, *n* (%)	8 (33)
Atrial fibrillation, *n* (%)	8 (33)
Hypertension, *n* (%)	21 (88)
Diabetes, *n* (%)	9 (38)
Smoking, *n* (%)	4 (17)
Admission NIHSS, median (IQR)	9 (7–13)
(pC)-ASPECTS, mean (IQR)	9 (8–9)
Time-from-onset-to-groin, mean (IQR), min	183 (112–245)
M3 segment, *n* (%)	11 (46)
M4 segment, *n* (%)	1 (4)
A2 segment, *n* (%)	5 (3)
P2 segment, *n* (%)	5 (21)
P3 segment, *n* (%)	1 (4)
SCA, *n* (%)	1 (4)
Intravenous thrombolysis, *n* (%)	13 (54)
Large artery atherosclerosis, *n* (%)	10 (42)
Cardioembolic, *n* (%)	13 (54)
Undetermined etiology, *n* (%)	1 (4)
General anesthesia	24 (100)

NIHSS: National Institutes of Health Stroke Scale; IQR: interquartile ranges; SCA: superior cerebral artery.

### Outcomes

Patients who were treated with the SPORNS technique had complete or near complete recanalization (mTICI 2c/3) in 22 of 24 cases (92%). NIHSS at discharge decreased to a median of three (IQR 1–5), 13 (54%) had good functional outcomes, and 18 (75%) had excellent functional outcomes defined as mRS 0-1 or mRS 0-2, respectively.

Two patients had small subarachnoid hemorrhages (8%) at follow-up CT, and two patients (8%) had symptomatic intracerebral hemorrhage; interestingly both of them had received additional intravenous tPA. No embolisms to new territories (ENT) occurred and the mean ASPECTS at follow-up was eight (7–9) with 13 (54%) patients showing a partial infarct on follow-up imaging in the affected territory (Table 2).

**Table 2.** Procedural, clinical, and functional outcome characteristics.

Outcome variables	All (*n* = 24)
Final rate of mTICI 3, *n* (%)	14 (58)
Final rate of mTICI 2c/3, *n* (%)	22 (92)
Total number of attempts, median (IQR)	1 (1–2)
Discharge NIHSS, median (IQR)	3 (1–5)
mRS at 90d, median (IQR)	1 (1–2)
mRS at 90d, 0-1, *n* (%)	13 (54)
mRS at 90d, 0-2, *n* (%)	18 (75)
mRS at 90d, 0, *n* (%)	6 (25)
mRS at 90d, 1, *n* (%)	7 (29)
mRS at 90d, 2, n (%)	5 (21)
mRS at 90d, 3, *n* (%)	3 (12)
mRS at 90d, 4, *n* (%)	2 (8)
mRS at 90d, 5, *n* (%)	0 (0)
mRS at 90d, 6, *n* (%)	1 (4)
Mortality at day 90, *n* (%)	1 (4)
Subarachnoid hemorrhage, *n* (%)	2 (8)
Symptomatic intracerebral hemorrhage, *n* (%)	2 (8)
Embolization to new territory, *n* (%)	0
ASPECTS 48 h, mean (IQR)	8 (7–9)
Time-from-groin-to-recanalization, mean (IQR), min	44 (32–58)

mTICI: modified treatment in cerebral ischemia; NIHSS: National Institutes of Health Stroke Scale; IQR: interquartile ranges; mRS: modified Rankin scale.

## Discussion

Our study shows that our initial experience of thrombectomy employing the SPORNS technique in stroke due to very distal vessel occlusion is safe and effective resulting in high rates of complete and near complete recanalization with very low rates of subarachnoid hemorrhage.

**Figure 2.**
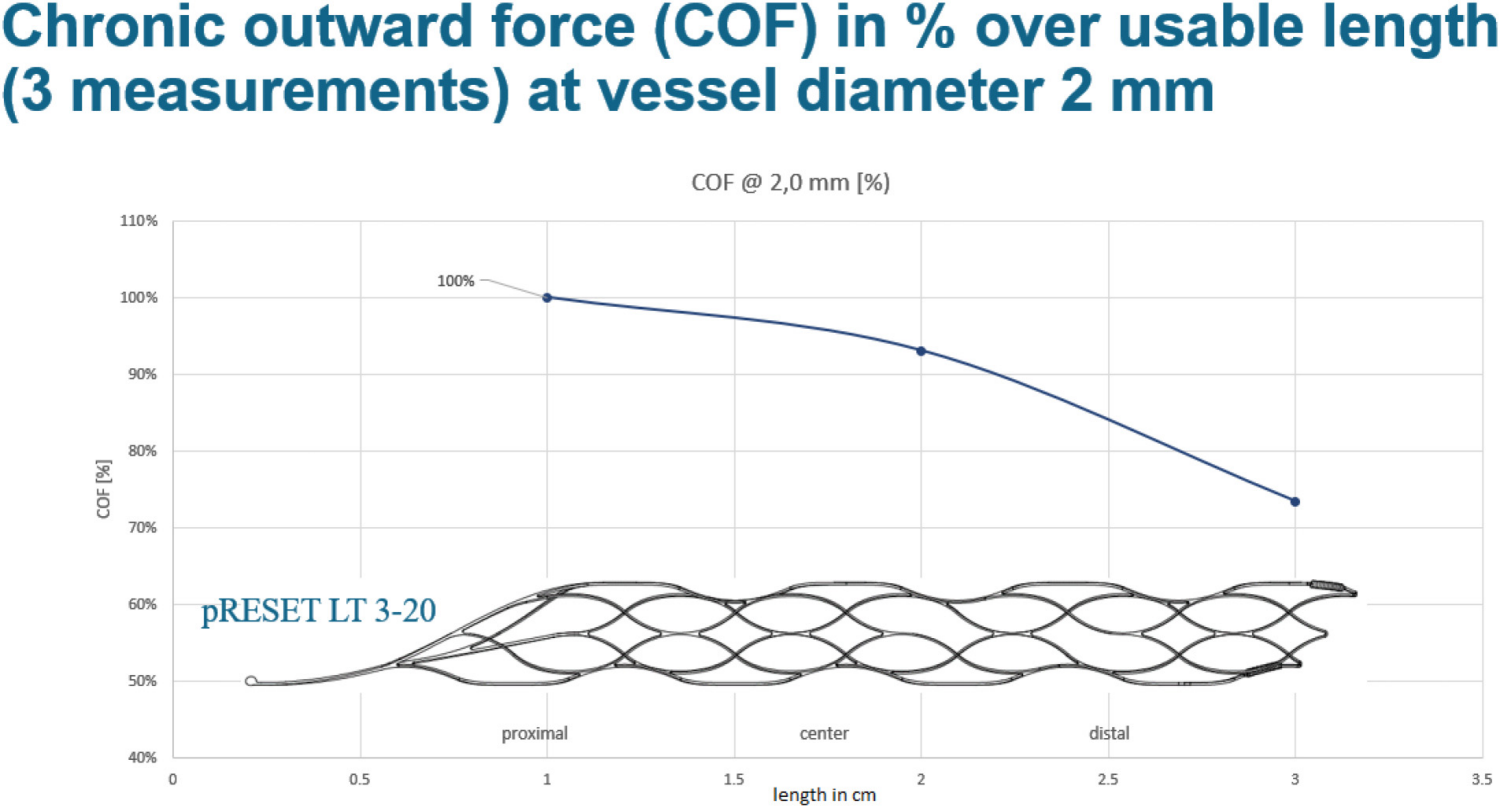
Illustration of the chronic outward force of Phenox pRESET LT 3–20 stent retriever at vessel diameter 2 mm. All sizes of the Phenox stent retriever (pRESET 4 × 20 mm, pRESET-lite 4 × 20 mm, and pRESET-lite 3 × 20 mm) have a maximum of 100% of radial force at the proximal end of the stent retriever and only 70% of radial force at the distal end. This allows for a reduction of the radial force and thus shearing on the vessel with only partial unsheathing using the soft partial release of the non-aggressive stent retriever (SPORNS) technique. Of note, the pRESET-lite 3–20 has a “working length” of 20 mm but the “shaft length” is 30 mm.

The smaller caliber of more fragile distal arteries and the more distal, thus harder to reach, occlusion location compared with LVOs warrant changes in EVT technique and technology to minimize risks. Only initial reports on the best technique and outcomes after EVT for very distal occlusions exist.^9^ For example the recently described microcatheter aspiration technique was reported to have a good safety profile but a moderate recanalization rate of 54% (defined as mTICI ≥ 2b). The main likely reason for the reduced risk of subarachnoid hemorrhages of the SPORNS technique may be the partially deployed stent retriever causing less friction and pulling force on soft distal arterial branches and perforating arteries.^10–12^ Furthermore, there is less tendency of straightening the anatomic curvature and loops of distal arteries resulting in less shear force on penetrating cortical arterioles. The partial release of the stent-retriever not only creates less pulling stress on vasculature, but also the microcatheter remains distal and may serve as an additional scaffolding structure for curved vasculature compared to the bare metal of the pusher wire in cases where the microcatheter is totally removed prior to clot retrieval. Additionally, the employed stent-retriever has a design feature that minimizes the radial force towards the tip. It is designed in a way that the maximum radial force is at the proximal part, while the radial force continuously decreases towards the distal tip as opposed to other stent retrievers where the radial force is the same over the whole stent retriever length or where this decrease is less pronounced (Figure 2). Thus, the partial release of this stent retriever further substantially reduces radial force over the deployed length.

The potentially higher efficacy of stent-retriever compared to aspiration catheter alone in the treatment of DVO has been reported previously^14,15^ and may be explained by smaller inner diameters of very small aspiration catheters (and especially microcatheters) which limits the possible vacuum to the clot and thus the efficacy of aspiration alone. Hence, the SPORNS technique for thrombectomy of very distal occlusions is a very promising approach combining the advantages of a highly efficacious stent-retriever technique with an improved safety profile. This technique should be explored further in multicenter and prospective studies and the upcoming distal thrombectomy randomized trials.^16^

Of note, anesthesia is an important point for thrombectomy of very distal occlusions as—in contrast to thrombectomy for large vessel occlusion strokes^17^—exact navigation of very distal vessels should be performed under general anesthesia. All patients were treated under general anesthesia in this study.

A limitation of our initial study is the retrospective design, the low number of included patients, and the lack of a control group. However, our observations are supported by in-vitro evidence and only preliminary studies about the best thrombectomy technique for very distal arterial occlusions exist.^9,16,18,19^ The observed excellent safety profile of our proposed technique compared to scarce published data on DVO treatment warrants further investigation.^9,16^

## Conclusion

Our study shows that the SPORNS technique with a soft partial release of a non-aggressive stent retriever is a promising technique for thrombectomy of very distal arterial occlusions. We found an excellent safety profile concurrent with high rates of complete or near complete recanalization resulting in mostly favorable outcomes. This preliminary evidence needs to be confirmed by larger-scale prospective studies.
